# The Effects of Iloprost on Oxygenation During One-Lung Ventilation for Lung Surgery: A Randomized Controlled Trial

**DOI:** 10.3390/jcm8070982

**Published:** 2019-07-05

**Authors:** Hoon Choi, Joonpyo Jeon, Jaewon Huh, Jungmin Koo, Sungwon Yang, Wonjung Hwang

**Affiliations:** 1 Department of Anesthesia and Pain Medicine, Seoul St. Mary’s Hospital, College of Medicine, The Catholic University of Korea, 222, Banpo-daero, Seocho-gu, Seoul 06591, Korea; 2 Department of Anesthesia and Pain Medicine, Eunpyeong St. Mary’s Hospital, College of Medicine, The Catholic University of Korea, 93-6, Jingwan-dong, Eunpyeong-gu, Seoul 122200, Republic of Korea

**Keywords:** iloprost, prostaglandins, one-lung ventilation, hypoxia, anesthesia, general

## Abstract

Hypoxemia can occur during one-lung ventilation (OLV) in thoracic surgery, leading to perioperative complications. Inhaled iloprost is a selective pulmonary vasodilator with efficacy in patients with pulmonary hypertension. The purpose of this study was to evaluate the effects of off-label inhaled iloprost on oxygenation during OLV in patients undergoing lung surgery. Seventy-two patients who were scheduled for elective video-assisted thoracoscopic lobectomy were assigned to receive an inhaled nebulizer of distilled water (control group), 10 μg iloprost (IL10 group), or 20 μg iloprost (IL20 group). Arterial and venous blood gas and hemodynamic analyses were obtained. Changes in partial pressure of oxygen in arterial blood (PaO_2_), after the initiation of OLV and the resumption two-lung ventilation (TLV), were similar in all three groups. However, PaO_2_ in the IL10 group was comparable to that in the control group, whereas PaO_2_ in the IL20 group was significantly higher than that in the control group at 10, 20, and 30 min after administration of iloprost (275.1 ± 50.8 vs. 179.3 ± 38.9, *p* < 0.0001; 233.9 ± 39.7 vs. 155.1 ± 26.5, *p* < 0.0001; and 224.6 ± 36.4 vs. 144.0 ± 22.9, *p* < 0.0001, respectively). The shunt fraction in the IL20 group was significantly higher than that in the control group after administration of iloprost (26.8 ± 3.1 vs. 32.2 ± 3.4, *p* < 0.0001; 24.6 ± 2.2 vs. 29.9 ± 3.4, *p* < 0.0001; and 25.3 ± 2.0 vs. 30.8 ± 3.1, *p* < 0.0001, respectively). Administration of inhaled iloprost during OLV improves oxygenation and decreases intrapulmonary shunt.

## 1. Introduction

One-lung ventilation (OLV) (i.e., intentional collapse of the lung on the operative side) facilitates surgical exposure in thoracic surgery [1,2]. During OLV, hypoxemia can occur due to an intrapulmonary shunt, in turn resulting from the mixing of unoxygenated blood from the collapsed non-dependent lung with oxygenated blood from the still-ventilated dependent lung [1,2]. Hypoxemia during OLV has an incidence of approximately 5–10% and is associated with perioperative complications, such as myocardial ischemia, cognitive dysfunction, atrial fibrillation, renal failure, and pulmonary hypertension [1,3]. Therefore, it is important to prevent hypoxemia by reducing the shunt fraction during OLV to improve postoperative outcomes.

Iloprost, a stable prostacyclin analogue, dilates systemic and pulmonary arterial vascular beds. When iloprost is used as an inhalant for treating pulmonary hypertension, it preferentially acts on pulmonary vessels, lowering pulmonary vascular resistance (PVR) [4]. Previous studies have shown that inhaled iloprost was effective as a selective pulmonary vasodilator not only in cases of chronic obstructive pulmonary disease (COPD) and acute respiratory distress syndrome (ARDS) with pulmonary hypertension but even in normal healthy volunteers without pulmonary hypertension [4,5,6]. In addition, inhaled iloprost was better-distributed in the well-ventilated region and gravity-dependent area, which affected the shunt fraction by redistributing pulmonary blood flow [5]. The same principle can be applied during OLV; iloprost inhaled during OLV may result in selective pulmonary vasodilation on the ventilated side.

Therefore, we hypothesized that iloprost inhaled during OLV would improve oxygenation and decrease intrapulmonary shunt by redirecting pulmonary blood flow to the well-oxygenated dependent lung. The purpose of the present study was to evaluate the effects of inhaled iloprost on oxygenation during OLV in patients undergoing lung surgery.

## 2. Materials and Methods

This study was approved by the Institutional Review Board of Seoul St. Mary’s Hospital, The Catholic University of Korea, Seoul, Korea (KC14MISI0880). Written informed consent was obtained from all patients participating in the study. The study was retrospectively registered at ClinicalTrials.gov (NCT03936140). From January 4, 2018, to October 24, 2018, we enrolled non-small cell lung cancer (stage I) patients, aged 20–75 years and with American Society of Anesthesiologists physical status I–III, who were scheduled for elective video-assisted thoracoscopic lobectomy. Exclusion criteria were heart failure (New York Heart Association III–IV), clinically significant arrhythmia, pulmonary hypertension (mean pulmonary artery pressure >25 mmHg), end-stage organ disease (i.e., hepatic/renal failure), severe obstructive or restrictive patterns in preoperative pulmonary function tests, history of previous thoracic surgery, and inability to provide informed consent.

This study was a prospective, double-blind, balanced (1:1), randomized controlled parallel-group trial. No changes were made to the design or protocol during the course of the study. The patients were randomly assigned to one of three groups: control group (distilled water), IL10 group (iloprost 10 μg), or IL20 group (iloprost 20 μg). Randomization was performed using a computer-generated randomization sequence, by an investigator who was not involved in patient care. Allocation results were concealed in sealed opaque envelopes, which were given to an anesthesia nurse not involved in patient care or assessment. The nurse prepared the solution for inhaled nebulizers in identical 2 mL syringes according to the group allocation. Test (2 mL) solutions were prepared, containing only distilled water in the control group, 10 μg iloprost in the IL10 group, and 20 μg iloprost in the IL20 group. Patients, surgeons, attending anesthesiologists, and nursing staff were all blinded to the group assignment during the entire study.

On the evening before surgery, pulmonary function tests and arterial blood gas analysis (breathing room air) were performed in all patients. A central venous catheter was inserted into the right subclavian vein, and the location of tip of the catheter at the junction of the superior vena cava and the right atrium was confirmed by fluoroscopy.

Patients did not receive any premedication. Upon arrival to the operating room, routine monitoring, including electrocardiography, pulse oximetry, non-invasive blood pressure, and end-tidal carbon dioxide, were conducted, with data recorded every 5 min. Anesthesia was induced with target-controlled infusion of propofol and remifentanil. After loss of consciousness, rocuronium 0.8 mg/kg was administered to facilitate tracheal intubation with a left-sided double-lumen endobronchial tube (Broncho-cath; Mallinckrodt, Dublin, Ireland: 37F for males and 35F for females). Correct positioning of the tube was confirmed by flexible fiberoptic bronchoscopy in both the supine and lateral decubitus positions. Mechanical ventilation was initiated with 100% oxygen, a tidal volume of 6 mL/kg predicted body weight, I:E ratio of 1:2, and a respiratory rate sufficient to maintain partial pressure of carbon dioxide in the arterial blood (PaCO_2_) to within 35–45 mmHg. The same mechanical ventilation was used for OLV. If the airway pressure exceeded 25 cmH_2_O, the tidal volume was adjusted. The fraction of inspired oxygen (FiO_2_) was maintained at 1.0 throughout the surgery. Anesthesia was maintained by target-controlled infusion of propofol and remifentanil, with the bispectral index (A-2000TM SP; Aspect Medical Systems, Norwood, MA, USA) values held between 40–60 and the systolic blood pressure within 15% of baseline; neuromuscular block was maintained with atracurium 0.3 μg/kg/min. After the induction of anesthesia, an arterial catheter was inserted into the radial artery for arterial pressure monitoring and blood sampling.

After patient positioning and initiation of OLV, the test solution was administered for 5 min via a jet nebulizer (Aerotech II; Pharmalucence Inc., Bedford, MA, USA) connected to the Y-piece of the ventilator’s inspiratory line with an oxygen flow of 6 L/min. Based on previous data, we expected that this method of iloprost administration would result in a dose to the lung of 2.5 μg in the IL10 group and 5.0 μg in the IL20 group [7].

Arterial and venous blood gas analysis and hemodynamic variables were obtained at five time points: 15 min after changing the patient’s position to the lateral decubitus position with two-lung ventilation (TLV) (T1); 10, 20, and 30 min after administration of iloprost in the lateral decubitus position with OLV (T2, T3, and T4, respectively); and 15 min after resuming TLV in the lateral decubitus position (T5). We calculated the cardiac output and converted it to the cardiac index according to the Fick’s method. Occlusion of the pulmonary artery for the surgical procedure was done after T4.

The primary outcome of the study was the partial pressure of oxygen in arterial blood (PaO_2_) 30 min after administration of iloprost. The secondary outcomes included the shunt fraction, hemodynamic variables and arterial blood gas analysis parameters. Shunt fraction (Q_s_/Q_t_) was calculated using the following formula:

Q_s_/Q_t_ = (CcO_2_−CaO_2_)/(CcO_2_−CvO_2_), (1)

whereby:

CaO_2_ = (PaO_2_ × 0.0031) + (Hemoglobin × 1.36 × SaO_2_), (2)

CvO_2_ = (PvO_2_ × 0.0031) + (Hemoglobin × 1.36 × SvO_2_), (3)

CcO_2_ = ([FiO_2_ × (P_B_ − P_H2O_) − PaCO_2_/Respiratory quotient] × 0.0031) + (Hemoglobin × 1.36), (4)

where P_B_ is the barometric pressure (760 mmHg), the P_H2O_ is 47 mmHg, the respiratory quotient is 0.8, CcO_2_ is the pulmonary capillary blood oxygen content, CaO_2_ is the arterial oxygen content, CvO_2_ is the mixed venous oxygen content (in which right atrial blood sample was used instead of the conventional pulmonary arterial blood sample), PvO_2_ is the partial pressure of oxygen in the central vein, SaO_2_ is the arterial oxygen saturation, and SvO_2_ is the central venous oxygen saturation.

Based on our preliminary data, the expected difference in the PaO_2_ at T3 was 40 mmHg, with an expected standard deviation of 45 mmHg. A sample size of 20 participants per group was needed to achieve a power of 0.8 and an α level of 0.05. Assuming a 20% dropout rate, we enrolled 24 patients in each group.

All values are expressed as means ± standard deviation, medians (interquartile range), or numbers (proportion), as appropriate. The Shapiro-Wilk test was used to check the normality of the data distribution; normally distributed data were analyzed using one-way ANOVA. Non-normally distributed or nonparametric data were analyzed using the Kruskal-Wallis test. Continuous variables were analyzed using ANOVA for repeated measures, and by one-way ANOVA if a significant difference was found among the groups. Paired t-tests with Bonferroni correction for multiple comparisons were performed to identify any significant changes from baseline values. Pearson’s chi-squared test was used for analyzing categorical data. No interim analyses were performed. A *p*-value of <0.05 was considered statistically significant. The statistical analysis was done using SPSS for Windows software (ver. 24.0; SPSS Inc., Chicago, IL, USA).

## 3. Results

**Figure 1:**
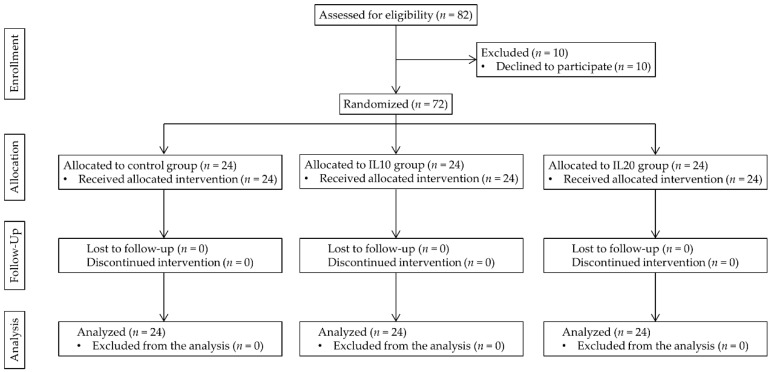
Consolidated Standards of Reporting Trials (CONSORT) flowchart of the study.

In total, 82 patients were assessed for eligibility, and 72 were enrolled in the study. All patients completed the study, and there were no missing or lost data. The study flow chart is presented in Figure 1.

Patient characteristics and preoperative test results, including the pulmonary function test and arterial blood gas analysis, were comparable among all three groups (Table 1). Intraoperative data, including total propofol and remifentanil consumption and the duration of surgery and anesthesia, were also comparable among all three groups (Table 2).

**Figure 2:**
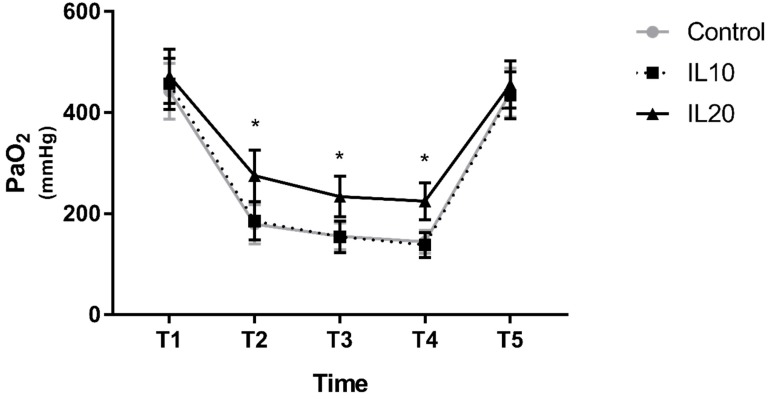
The PaO_2_. Error bars represent standard deviation. IL10, iloprost 10 μg group; IL20, iloprost 20 μg group; * indicates *p* < 0.0001 compared to the control group. T1, 15 min after changing the patient’s position to the lateral decubitus position with two-lung ventilation (TLV); T2, T3, T4, 10, 20, and 30 min after administration of iloprost in the lateral decubitus position with one-lung ventilation (OLV), respectively; and T5, 15 min after resuming TLV in the lateral decubitus position.

**Figure 3:**
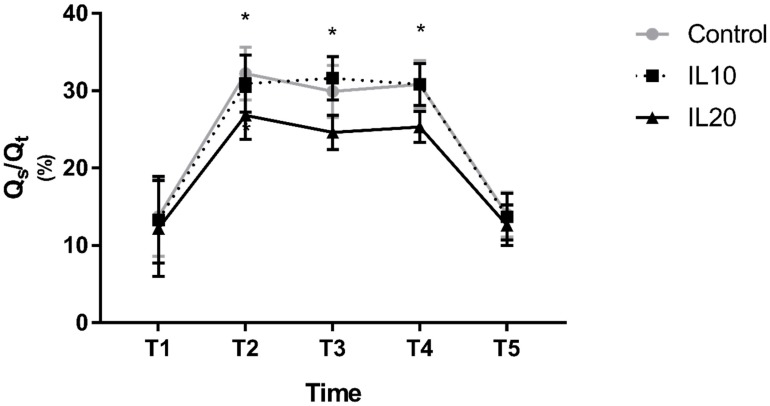
Shunt fraction. Error bars represent standard deviation. * indicates *p* < 0.0001 compared to the control group. T1, 15 min after changing the patient’s position to the lateral decubitus position with TLV; T2, T3, T4, 10, 20, and 30 min after administration of iloprost in the lateral decubitus position with OLV, respectively; and T5, 15 min after resuming TLV in the lateral decubitus position.

Changes in PaO_2_ after the initiation of OLV, and after resuming TLV, were similar among all three groups; compared to T1, PaO_2_ decreased significantly at T2, T3, and T4 and was comparable at T5. However, the degree of decrease in PaO_2_ at T2, T3, and T4 differed among the three groups. PaO_2_ in the IL10 group was comparable to that in the control group at T2, T3, and T4, whereas PaO_2_ in the IL20 group was significantly higher than that in the control group at T2, T3, and T4 (275.1 ± 50.8 vs. 179.3 ± 38.9, *p* < 0.0001 at T2; 233.9 ± 39.7 vs. 155.1 ± 26.5, *p* < 0.0001 at T3; and 224.6 ± 36.4 vs. 144.0 ± 22.9, *p* < 0.0001 at T4; Figure 2). Q_s_/Q_t_ showed similar results. Compared to T1, Q_s_/Q_t_ decreased significantly at T2, T3, and T4 and was comparable at T5 in all three groups. Q_s_/Q_t_ in the IL10 group was comparable to that in the control group during OLV. However, Q_s_/Q_t_ in the IL20 group was significantly lower than that in the control group (26.8 ± 3.1 vs. 32.2 ± 3.4, *p* < 0.0001 at T2; 24.6 ± 2.2 vs.29.9 ± 3.4, *p* < 0.0001 at T3; and 25.3 ± 2.0 vs. 30.8 ± 3.1, *p* < 0.0001 at T4; Figure 3).

Other values derived from arterial and vein blood gas samples, including SaO_2_, were comparable among all three groups at all time periods. Hemodynamic parameters, including heart rate and mean arterial blood pressure (MBP), were also comparable among all three groups at all time periods (Table 3).

## 4. Discussion

The aim of the present study was to demonstrate the effects of inhaled iloprost on oxygenation during OLV in lung surgery. The results suggested that the degree of intrapulmonary shunt was reduced after the administration of inhaled iloprost, which resulted in improved oxygenation. Although a 10 μg dose of iloprost did not improve oxygenation nor the degree of shunt, a 20 μg dose of iloprost did improve oxygenation and the degree of shunt.

To our knowledge, this study is the first to evaluate the effects of inhaled iloprost on oxygenation in patients receiving general anesthesia. Previous studies have examined the effects of inhaled iloprost only in patients not receiving anesthesia. Inhaled iloprost resulted in improved gas exchange, without any detrimental effects on pulmonary mechanics nor systemic hemodynamics in ARDS and COPD patients with pulmonary hypertension [4,6]. However, in other studies on patients with COPD and pulmonary hypertension, inhaled iloprost not only failed to improve oxygenation and exercise capacity but, in fact, worsened oxygenation in patients with mild to moderate pulmonary hypertension [8,9]. In studies that demonstrated an improvement, the authors assumed that iloprost was delivered predominantly to the well-ventilated areas of the lung, thus preferentially vasodilating those areas and resulting in decreased ventilation/perfusion (V/Q) mismatch. On the other hand, in studies that failed to show improvement, delivery of iloprost to the well-ventilated areas might have failed, thus counteracting hypoxic pulmonary vasoconstriction (HPV) and increasing V/Q mismatch. The authors also hypothesized that air trapping may have occurred, caused by the broncho-dilating properties of iloprost and respiratory depression owing to decreased cerebral blood flow.

In our study, iloprost was selectively administered to the ventilated lung through a double-lumen endobronchial tube. Therefore, improved oxygenation was achieved by selective vasodilation of the ventilated lung and avoiding interference with HPV in the non-ventilated lung, which led to a favorable redistribution of blood flow in the lung. The likelihood of air trapping or respiratory depression in our study was low, since the patients did not have severe respiratory diseases and were under mechanical ventilatory care. Moreover, in healthy volunteers with normal pulmonary vascular tone, inhaled iloprost has been shown to result in a more gravity-dependent pulmonary perfusion [5]. This might have contributed to the improved oxygenation observed in the present study, since lung surgery is performed in the lateral decubitus position with the ventilated lung being the dependent lung.

Hypoxemia during OLV is defined as SaO_2_ less than 90% at FiO_2_ greater than 0.5, or PaO_2_ less than 60 mmHg at FiO_2_ of 1.0 [1,2]. Hypoxemia occurs due to the perfusion of the collapsed, nonventilated lung, leading to transpulmonary shunting and impairment in oxygenation. Although the degree might be different, the impairment in oxygenation is inevitable during OLV. The decrease in PaO_2_ during OLV compared to TLV in this study was consistent with previous studies of oxygenation during OLV. The trend of decrease in PaO_2_ during the first 30 min of OLV in this study was also consistent with previous studies [10,11,12]. The increase in atelectasis during positive pressure ventilation and delayed effect of HPV, which has a biphasic nature and its second phase effect occurring approximately 40 min after initiation of OLV, are known to contribute to this natural phenomenon [13].

In the past, measures to prevent and treat hypoxemia during OLV have focused on airway complications and ventilation strategies. More recently, modulation of pulmonary perfusion to improve V/Q mismatch has been an area of interest; i.e., not interfering with HPV in the operative lung and minimizing PVR in the dependent lung, thus redirecting pulmonary blood flow to the well-oxygenated dependent lung [3,13].

Previous studies have investigated the use of inhaled vasodilators, such as nitric oxide (NO) and prostacyclin, which induce selective pulmonary vasodilation in the ventilated lung. In patients with COPD and pulmonary hypertension, inhaled NO has been shown to improve oxygenation and pulmonary hemodynamics without any detrimental effect upon systemic hemodynamics [14,15]. However, administration of NO to the ventilated lung during OLV has not been shown to improve oxygenation [16,17], with limited effects in patients with severe pulmonary hypertension [16] or when used in combination with almitrine [18]. There are also other disadvantages; for example, a specific delivery system is required, and inhaled NO has pro-inflammatory effects and can result in oxidative injury and the nitrosylation of proteins [19,20]. Inhaled prostacyclins, such as epoprostenol, exhibit effects on the pulmonary vasculature similar to those of inhaled NO [14]. In a case report of a patient with decreased diffusing capacity, inhaled epoprostenol in combination with systemic phenylephrine improved oxygenation during OLV [21]. However, epoprostenol is diluted in a glycine buffer that tends to adhere to ventilator valves, and its delivered concentration is therefore difficult to predict [22]. Other drugs used for pulmonary hypertension, such as sildenafil and riociguat, might have a role in modulation of pulmonary perfusion during OLV. However, application of these drugs during anesthesia is challenging [23].

The prostacyclin used in the present study was iloprost. Inhaled iloprost is an effective pulmonary vasodilator that improves oxygenation, even in patients with normal PVR [4,5,6]. Its delivery is simple, cost-effective, and predictable compared to NO and epoprostenol [22]. Recently, new devices and formulations have been introduced to increase drug deposition and plasma levels of iloprost while shortening inhalation time [24,25,26]. Currently, application of these methods in the operating room setting during anesthesia is challenging. However, in the future, the delivery of iloprost via these new methods applied to the anesthesia machine might provide a more effective and simpler route. Therefore, we recommend inhaled iloprost as a simple and safe method to improve oxygenation during OLV. Moreover, in patients with reduced diffusing capacity and impaired gas exchange at risk of hypoxemia during OLV, we expect that inhaled iloprost may be an effective measure for preventing hypoxemia, similar to epoprostenol. However, further studies are needed in these groups of patients to validate this.

The doses of iloprost used in the present study were 10 and 20 μg, diluted with distilled water as a 2 mL solution. These doses, when administered via a jet nebulizer on the inspiratory line of the ventilator, are known to result in delivered doses to the lung of 2.5 and 5.0 μg, respectively [7]. The 10 μg dose of iloprost did not improve oxygenation or the degree of shunt, while 20 μg iloprost improved both outcomes. The patient population in this study may have contributed to this finding: All patients had normal PVR. In patients with elevated PVR, using the same type of nebulizer, doses of 10 μg iloprost have previously resulted in maximal efficacy [4]. Conversely, in normal healthy volunteers without pulmonary hypertension (i.e., a patient population similar to that in the present study), a 20 μg dose of iloprost was required before effects were observed [7].

A major side effect of prostacyclin is inhibition of platelet activation [4]. The inhibition of platelet activation can be a concern in patients undergoing lung surgery. However, inhaled prostacyclin has been reported to be safe in patients after cardiothoracic surgery [22]. In this study, none of the patients needed transfusion of red blood cell, and none showed significant blood loss or postoperative coagulation dysfunction.

The present study had several limitations. First, delivery of inhaled iloprost is more efficient with ultrasonic nebulizers [27]. However, ultrasonic nebulizers are not compatible with anesthesia machines; therefore, a jet nebulizer compatible with the anesthesia machine was used in this study. Although the jet nebulizer requires a longer duration of inhalation and is considered less efficient than the ultrasonic nebulizer, the method of delivery has been validated in other studies [4,7]. Second, right atrial blood samples were used to calculate the shunt instead of pulmonary arterial blood samples. Moreover, the effect of inhaled iloprost on ventilated lungs may have been more accurately determined if measurements of pulmonary artery pressure in both lungs were taken. However, pulmonary artery catheters are not routinely used in lung surgery because of their invasive nature. Also, previous studies have shown that pulmonary arterial blood samples can be substituted by right atrial blood samples [10,12,28,29]. Third, inhaled iloprost might result in air trapping and exacerbate pre-existing V/Q mismatch [8,9], but these were not explored in this study. Currently, the anesthesia machine cannot measure auto-positive end-expiratory pressure (autoPEEP), and therefore it cannot detect air trapping. Also, a V/Q scan is not considered a routine preoperative test. However, patients with severe obstructive or restrictive patterns in preoperative pulmonary function tests were excluded from the study, and therefore the likelihood of air trapping and pre-existing V/Q mismatch was low. Fourth, the patients enrolled in the present study had relatively normal pulmonary function, and therefore the risk of hypoxemia from OLV was minimal. We excluded patients with pulmonary hypertension and severe abnormalities in the preoperative pulmonary function test. Although iloprost showed improvement in oxygenation, the patients without administration of iloprost did not show clinically significant hypoxemia. However, as in the case of inhaled NO, patients with pulmonary hypertension or an abnormal pulmonary function test are expected to have more benefit from inhaled iloprost [16]. Finally, we did not measure the plasma level of iloprost in this study.

In conclusion, administration of inhaled iloprost during OLV improves oxygenation and decreases intrapulmonary shunt. Iloprost inhalation via the jet nebulizer has the potential to prevent hypoxemia in patients requiring OLV for thoracic surgery. Further validation is needed in high-risk patients.

## Figures and Tables

**Table 1:** Preoperative characteristics.

	Control Group (*n* = 24)	IL10 Group (*n* = 24)	IL20 Group (*n* = 24)	*p* Value
Age (year)	60 ± 9	57 ± 7	58 ± 8	0.504
Sex (Male/Female)	15/9	13/11	15/9	0.794
Height (cm)	163.7 ± 8.1	162.2 ± 9.5	166.2 ± 6.8	0.220
Weight (kg)	62.7 ± 6.0	59.4 ± 6.3	59.4 ± 6.1	0.111
ASA class (I/II/III)	4/15/5	2/16/6	2/19/3	0.641
Operation side (Right/Left)	16/8	14/10	16/8	0.786
Pulmonary function test				
FEV_1_ (% predicted)	93 ± 13	91 ± 15	93 ± 11	0.750
FVC (% predicted)	94 ± 9	92 ± 6	93 ± 6	0.618
FEV_1_/FVC (%)	75 ± 10	74 ± 9	79 ± 10	0.203
Arterial blood gas analysis				
pH	7.43 ± 0.04	7.43 ± 0.05	7.42 ± 0.05	0.596
PaCO_2_ (mmHg)	37.6 ± 3.2	39.2 ± 3.2	37.1 ± 4.4	0.131
PaO_2_ (mmHg)	97.6 ± 9.5	98.9 ± 5.7	99.6 ± 9.6	0.709
SaO_2_ (%)	97.5 ± 1.4	97.2 ± 1.4	97.3 ± 1.2	0.633

The data are presented as the mean ± standard deviation. FEV_1_, forced expiratory volume (1 s); FVC, forced vital capacity; PaCO_2_, partial pressure of carbon dioxide in the arterial blood; PaO_2_, partial pressure of oxygen in the arterial blood; SaO_2_, arterial oxygen saturation.

**Table 2:** Intraoperative characteristics.

	Control Group (*n* = 24)	IL10 Group (*n* = 24)	IL20 Group (*n* = 24)	*p* Value
Total propofol use (mg)	1693 ± 475	1929 ± 662	1850 ± 467	0.314
Total remifentanil use (μg)	1654 ± 356	1906 ± 798	1749 ± 556	0.343
Duration of surgery (min)	163 ± 43	177 ± 60	193 ± 60	0.179
Duration of anesthesia (min)	209 ± 42	223 ± 63	239 ± 58	0.188

The data are presented as the mean ± standard deviation.

**Table 3:** Arterial/vein blood gas and hemodynamic analysis.

	T1	T2	T3	T4	T5	*p* Value
pH						0.826
Control	7.43 ± 0.06	7.43 ± 0.06	7.42 ± 0.04	7.41 ± 0.04	7.41 ± 0.04	
IL10	7.44 ± 0.06	7.43 ± 0.05	7.42 ± 0.05	7.41 ± 0.05	7.40 ± 0.04	
IL20	7.41 ± 0.06	7.44 ± 0.05	7.42 ± 0.04	7.41 ± 0.04	7.41 ± 0.07	
PaCO_2_ (mmHg)						0.830
Control	37.1 ± 3.7	38.2 ± 2.4	38.3 ± 1.9	38.4 ± 2.5	38.0 ± 2.4	
IL10	36.5 ± 3.7	38.1 ± 3.2	38.7 ± 2.9	38.6 ± 2.6	37.8 ± 1.9	
IL20	36.9 ± 3.2	37.9 ± 2.6	38.3 ± 1.7	38.5 ± 3.1	37.6 ± 2.4	
SaO_2_ (%)						0.362
Control	98.7 ± 0.7	98.2 ± 0.8	98.7 ± 0.7	98.5 ± 0.5	98.7 ± 0.4	
IL10	98.8 ± 0.6	97.9 ± 0.9	98.7 ± 0.6	98.8 ± 0.5	98.7 ± 0.4	
IL20	98.7 ± 0.6	98.2 ± 0.8	98.9 ± 0.4	98.8 ± 0.6	98.7 ± 0.5	
PvO_2_ (mmHg)						0.918
Control	49.8 ± 5.0	48.5 ± 3.6	47.8 ± 7.2	48.5 ± 8.3	50.9 ± 8.3	
IL10	50.9 ± 8.7	46.7 ± 6.2	47.8 ± 4.8	47.9 ± 5.4	50.9 ± 5.0	
IL20	50.9 ± 6.3	47.7 ± 5.7	46.8 ± 4.5^§^	49.5 ± 5.5	51.2 ± 5.4	
SvO_2_ (%)						0.986
Control	80.0 ± 3.4	77.4 ± 5.3^§^	77.6 ± 3.8^§^	76.9 ± 3.4^§^	79.8 ± 4.2	
IL10	79.1 ± 3.2	76.9 ± 3.3^§^	77.2 ± 3.7	76.7 ± 2.5^§^	79.3 ± 2.9	
IL20	80.4 ± 3.2	78.3 ± 3.4^§^	77.4 ± 2.1^§^	76.6 ± 3.3^§^	79.9 ± 3.9	
Hemoglobin (g/dL)						0.482
Control	11.9 ± 1.3	11.8 ± 1.2	11.7 ± 1.1	11.6 ± 1.2	11.6 ± 1.1	
IL10	12.0 ± 1.0	11.8 ± 1.0	11.7 ± 1.2	11.6 ± 1.2	11.6 ± 1.3	
IL20	11.7 ± 1.4	11.4 ± 1.2	11.3 ± 1.2	11.2 ± 1.2	11.1 ± 1.2	
Heart rate (beat/min)						0.999
Control	64 ± 12	65 ± 6	66 ± 7	65 ± 8	65 ± 9	
IL10	63 ± 13	64 ± 11	66 ± 6	65 ± 7	65 ± 9	
IL20	64 ± 8	65 ± 10	66 ± 6	66 ± 7	65 ± 9	
MBP (mmHg)						0.762
Control	71 ± 10	78 ± 8	79 ± 7	80 ± 7	79 ± 7	
IL10	73 ± 9	76 ± 9	77 ± 11	81 ± 12	81 ± 9	
IL20	70 ± 8	76 ± 11	77 ± 11	78 ± 9	76 ± 10	
Cardiac Index (L/min/m^2^)						0.084
Control	4.4 ± 0.9	4.0 ± 0.9	3.9 ± 0.6	3.8 ± 0.6	4.6 ± 1.6	
IL10	4.1 ± 0.7	3.9 ± 0.6	3.9 ± 0.7	3.8 ± 0.6	4.3 ± 0.8	
IL20	4.5 ± 1.0	4.3 ± 0.8	3.9 ± 0.6	3.7 ± 0.5	4.7 ± 1.2	

The data are presented as the mean ± standard deviation. PvO_2_, partial pressure of oxygen in the central vein; SvO_2_, central vein oxygen saturation; MBP, mean arterial blood pressure; T1, 15 min after changing the patient’s position to the lateral decubitus position with TLV; T2, T3, T4, 10, 20, and 30 min after administration of iloprost in the lateral decubitus position with OLV, respectively; and T5, 15 min after resuming TLV in the lateral decubitus position.
